# Incorporating Spatial Statistics into Examining Equity in Health Workforce Distribution: An Empirical Analysis in the Chinese Context

**DOI:** 10.3390/ijerph15071309

**Published:** 2018-06-22

**Authors:** Bin Zhu, Chih-Wei Hsieh, Yue Zhang

**Affiliations:** 1 School of Public Policy and Administration, Xi’an Jiaotong University, 28 Xianning West Road, Xi’an 710049, China; 2 Department of Public Policy, City University of Hong Kong, Tat Chee Avenue, Kowloon, Hong Kong, China; cwhsieh@cityu.edu.hk (C.H.); yzhang297-c@my.cityu.edu.hk (Y.Z.)

**Keywords:** health workforce, health equity, horizontal equity, Theil index, Moran’s I, China

## Abstract

Existing measures of health equity bear limitations due to the shortcomings of traditional economic methods (i.e., the spatial location information is overlooked). To fill the void, this study investigates the equity in health workforce distribution in China by incorporating spatial statistics (spatial autocorrelation analysis) and traditional economic methods (Theil index). The results reveal that the total health workforce in China experienced rapid growth from 2004 to 2014. Meanwhile, the Theil indexes for China and its three regions (Western, Central and Eastern China) decreased continually during this period. The spatial autocorrelation analysis shows that the overall agglomeration level (measured by Global Moran’s I) of doctors and nurses dropped rapidly before and after the New Medical Reform, with the value for nurses turning negative. Additionally, the spatial clustering analysis (measured by Local Moran’s I) shows that the low–low cluster areas of doctors and nurses gradually reduced, with the former disappearing from north to south and the latter from east to west. On the basis of these analyses, this study suggests that strategies to promote an equitable distribution of the health workforce should focus on certain geographical areas (low–low and low–high cluster areas).

## 1. Introduction

Health equity is an important issue in health management and a crucial concern for policy makers, as it involves various aspects of the health discipline and heated exchanges are generated when health needs are confronted by resource constraints [1,2,3,4]. In practice, health workforce distribution pertains to the distribution and organization of members of the health workforce among health care departments or regions, which can reflect the degree of health equity [5].

It is widely recognized that health equity is commonly divided into horizontal and vertical dimensions; the former refers to equal treatment for people with equal needs, and the latter emphasizes different levels of treatments for people with unequal needs [6]. Almost all the theoretical or empirical studies concerning health equity, as well as the equity in health workforce distribution, have advocated the horizontal rather than the vertical dimension [7,8]. For instance, Kreng and Yang [7] defined an ideal distribution of health workforce as a condition where most people have equal access to health services despite geographical and/or economical differences. The World Health Organization (WHO) [9] also proposed accelerating progress toward achieving sustainable development goals by ensuring equitable access to the health workforce.

Existing methods for measuring equity for health workforce distribution include the Gini coefficient, Theil index, and Atkinson index [10], and each method has its own strengths. Specifically, the Gini coefficient is the most frequently used measurement of equity in health workforce distribution because it is simple to calculate and interpret when this approach is combined with the corresponding Lorenz curve [11,12]. Similar to the Gini coefficient, the Theil index can also be used to measure the overall degree of differences, but it uniquely shows the contributions within a subgroup and between subgroup components on the basis of the calculated contribution rate [13,14]. In contrast, the Atkinson index is particularly suitable for analyzing small differences and to determine which end of the sorted distribution contributes the most to the observed inequality [15].

The abovementioned methods vary but share commonalities—they are all designed to measure income inequality and then utilize this information for the measurement of horizontal equity in health workforce distribution [10]. However, income equality is slightly different from equity in terms of health workforce distribution. For instance, income equality is represented by the distribution of socioeconomic resources in a population, whereas equity in health workforce distribution refers to the distribution of the health workforce not only in a population but also in geographical units.

Differently put, the various spatial distributions of the same values in different administrative units are of little importance to the former but indicate a completely different situation for health workforce distribution. Therefore, existing methods for measuring the equity in health resource distribution have some shortcomings. First, the spatial distribution details are ignored as spatial location information is overlooked. For instance, the equal Gini coefficient value may correspond to hundreds of various kinds of spatial distribution patterns, which indicate different types of local characteristics. In addition, merely using traditional economic methods limits policy implications for policy makers due to their emphasis on quantitative differences. That is, these approaches primarily determine whether the health workforce is distributed equally rather than where the priority areas are. Therefore, the existing methods have limitations in providing evidence for policy makers.

By definition, the inequity in health workforce distribution is the spatial concentration of the health workforce, i.e., too many health workers in particular areas, but not enough in others [16]. Therefore, methods that can identify the concrete details of spatial distribution can also be considered by health scholars to understand the equity in health workforce distribution more effectively. As an emerging form of technology, spatial statistics focus on the distribution details and present excellent visualization by means of spatial data analysis software [17]. They have been widely used in epidemiology and health economics research [18,19,20,21]. The adoption of spatial statistics can be used to compensate for the limitations of traditional economic methods. More specifically, the combination of traditional economic methods and spatial statistics has two distinct advantages in measuring equity in health workforce distribution. First, these two methods complement each other. The traditional economic methods can be used for the measurement of global equity for an entire region, while spatial statistics help reveal the equity of health workforce distribution for local areas. Second, spatial statistics can identify the distinctive regions and/or administrative units, which tend to be the priority areas for promoting an equitable health workforce distribution.

To summarize, this study aims to evaluate the equity of health workforce distribution in China using traditional economic methods and spatial statistics. By doing so, this study adds value to existing literature and provides empirical evidence for health workforce planning in China. The rest of this paper is arranged as follows. Section 2 reviews China’s status quo of health workforce distribution and the reform process. Subsequently, methods and data are presented in Section 3. Section 4 displays the empirical results of traditional economic methods and spatial analysis. Finally, a discussion and conclusion are presented by summarizing the results and proposing policy recommendations.

## 2. Health Workforce Distribution in China

Health workforce misdistribution has been a major problem in China [22]. The shortage of the health workforce complicates the alleviation of the uneven distribution of the health workforce and leads to more policy being more focused on the total number of health workers rather than on regional resource allocation. It is the dual effect of resource constraints and policy priorities on total workforce size that results in the unbalanced distribution of the health workforce in China [23].

**Figure 1:**
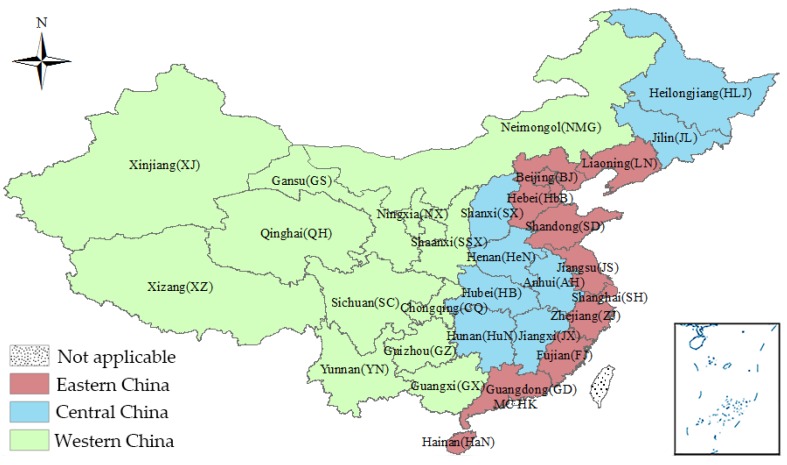
Regional divisions of mainland China (Province abbreviations are provided in parentheses).

As the largest developing country in the world, China covers a large territory and has many administrative units. According to National Health and Family Planning Yearbook, mainland China can be divided into eastern, central, and western regions on the basis of population, economy, and other factors (Figure 1) [24].

What has attracted scholars’ and policy makers’ attention to the health workforce distribution among these administrative units is the diversity at the provincial level. Specifically, the natural geographical advantages of its coastal location and the policy support make Eastern China dominate in the recruitment and retention of health workers. In contrast, Western China is disadvantaged in various aspects (e.g., the education, attraction, and retention of health workforce) due to its underdeveloped economy and unfavorable geographic position.

During the past decade China’s health system has undergone rapid development. The milestone in this period was the publication of the new scheme for medical care reform. In 2009, a new round of medical and health care system reforms (referred to as the New Medical Reform) officially began in mainland China. The reforms seek to enhance the performance of the health system and support underdeveloped areas. For this purpose, several policy guidelines were directly or indirectly committed to workforce building for health care because of the central role of the health workforce in the health system [25]. According to the China Health and Family Planning Statistical Yearbook, the total size of the health workforce increased dramatically after the official release of the New Medical Reform [24]. However, the growth of the total quantity of health workers may conceal underlying disparities; therefore, more systematic assessments that go beyond quantitative summary are necessary. To better evaluate the effects of the New Medical Reform, the equity in health workforce distribution in China will be discussed with regard to the regional divisions and the timeline. Three equidistant time points, 2004 (five years before the New Medical Reform), 2009, and 2014 (five years after the New Medical Reform) are selected to better display the spatial changing patterns of health workforce distribution in China.

## 3. Data and Methods

### 3.1. Data Resource and Indicators

The analysis centers on the provincial-level data in 2004, 2009, and 2014, which were obtained from China’s Health Statistical Yearbook and China’s Health and Family Planning Statistical Yearbook. Only the provincial administrative units in mainland China are included in this study because of data accessibility (please see the supplementary file Table S1 for the original data). It is worth noting that Hainan province was also excluded to ensure there is a neighboring unit in every province, which was required by the spatial analysis in this study.

As mentioned above, equality in economics is represented by the distribution of socioeconomic resources in a population. Actually, the essence of all the traditional economic methods is to compare the relative share of socioeconomic resources in a population. For example, the Lorenz curve plots cumulative resource shares relative to cumulative population shares; the shape of the Lorenz curve, which corresponds to the value of the Gini coefficient, is determined by the resource-to-population ratio in each administrative unit. Therefore, the amount of health workforce and population in each administrative unit is collected for the calculation of traditional economic methods.

The corresponding indicator for spatial analysis is the distribution of the health workforce in a population, which can also be referred to as health workforce density. It is usually expressed as the number of health workers per 1000 individuals, which is widely used in WHO reports, national policy documents, and academic studies to evaluate the performance of a health system [22,26,27]. Given that the health workforce in China varies and includes many types (e.g., doctors, nurses, pharmacists, and technicians), this study focuses on the groups of doctors (licensed doctors and licensed assistant doctors) and nurses (registered nurses with nursing certifications) because they directly provide health care services and account for approximately two-thirds of the total number of health workers [28].

### 3.2. Traditional Economic Method—Theil Index

Among all the traditional economic methods for measuring the equity in health resource distribution, only the Theil index can decompose the overall inequity into contributions within several groups and is exactly suitable for classification based on the regional divisions in China [10]. Hence, the Theil index was adopted to measure the overall equity of health workforce distribution in China and the contributions from each subregion.

Akin to the Gini coefficient, the value of the Theil index ranges from 0 to 1, and a smaller value means a more equitable condition. It can be calculated as follows:

(1) $$Theil\;index=\overset{n}{\underset{i=1}{\sum }}P_{i}ln\frac{P_{i}}{Y_{i}}$$

$P_{i}$: proportion of population in one area accounting for the total population;
$Y_{i}$: proportion of health workforce in one area accounting for the total health workforce.

If the areas are divided into several groups (e.g., k groups in the formula, Western, Central, and Eastern China in this case), the Theil index can also be decomposed into the $T_{intra-class}$ and $T_{inter-class}$ [10]. The contribution rates within and between groups can be calculated by dividing Theil index as the proportion of $T_{intra-class}$ and $T_{inter-class}$ accounted for. In this study, the higher proportion of $T_{inter-class}$ indicates that the inequity in health workforce distribution results more from the inter-regional difference between Western, Central and Eastern China, and vice versa.

(2) $$\begin{array}{c} Theil\;index=T_{intra-class}+T_{inter-class}\\ T_{intra-class}=\overset{k}{\underset{g=1}{\sum }}P_{g}T_{g}\\ T_{inter-class}=\overset{k}{\underset{g=1}{\sum }}P_{g}ln\frac{P_{g}}{Y_{g}} \end{array}$$

$T_{intra-class}$: degree of equity in health workforce distribution within the group.
$T_{inter-class}$: degree of equity in health workforce distribution between the groups.
$T_{g}$: Theil index of health workforce distribution in subgroups.
$P_{g}$: proportion of population in one group accounting for the total population.
$Y_{g}$: proportion of health workforce in one group accounting for the total health workforce.

### 3.3. Spatial Statistics

This study adopts spatial autocorrelation analysis due to its theoretical and practical implications for equity in health workforce distribution. One dimension of health inequity is the absence of systematic differences among geographically defined population groups or subgroups [29]. Spatial autocorrelation, which refers to the correlation among values of a single variable strictly attributable to the proximity of the values in geographical space, particularly targets the health inequity resulting from geographical locations [30]. Moran’s I is one of the most common indicators for measurement in spatial autocorrelation and has two forms—Global Moran’s I and Local Moran’s I [31]. The former reveals the spatial correlations of the whole region, whereas the latter can be regarded as the decomposition of the Global Moran’s I and reveals the spatial correlations of one specific area and its neighbors.

#### 3.3.1. Global Moran’s I

Global Moran’s I statistics, which reveal the relationship of attributes between adjacent areas are widely used in spatial studies to explore the existence of spatial autocorrelation [32,33]. Global Moran’s I ranges from −1 to 1 and resembles the Pearson correlation coefficient in interpretation. If a Global Moran’s I is above zero, it indicates a positive spatial autocorrelation (concentration tendency of similar values, high with high and low with low; the classification of high and low values depends on the mean value), and a higher value indicates stronger correlations. If a Global Moran’s I is less than zero, it indicates a negative correlation (concentration tendency of dissimilar values, high with low). In particular, zero means that all the high and low values are randomly distributed in space [34]. Global Moran’s I is defined as follows:

(3) $${\mathrm{Global}\;\mathrm{Moran}}^{\prime }I=\frac{n\sum_{i=1}^{n}\sum_{j=1}^{n}W_{ij}\left(y_{i}-\bar{y}\right)\left(y_{j}-\bar{y}\right)}{\left(\sum_{i=1}^{n}\sum_{j=1}^{n}W_{ij}\right)\sum_{i=1}^{n}{\left(y_{i}-\bar{y}\right)}^{2}}$$

In the formula, $y_{i}$ refers to the health workforce density, which is measured in the form of workforce-to-population ratio, and $\bar{y}$ is its mean value. *W* is a spatial weight matrix that shows the relationship among different units, i.e., contiguous or not. This study adopts a commonly used binary contiguity matrix. Specifically, if administrative units *i* and *j* are adjacent to each other, then the matrix element $W_{ij}$ = 1; otherwise, $W_{ij}$ = 0. By convention, the spatial matrix is normalized by line to eliminate the effects of the number of adjacent units.

#### 3.3.2. Spatial Classification

To visually display the details of spatial autocorrelation, this study uses Moran’s I scatterplots to reveal the relationship between the health workforce density for each area and the average values for its neighbors. In Moran’s I scatterplots, each spot denotes one administrative unit, and the horizontal and vertical axes represent the health workforce density in one administrative unit and the corresponding spatial lag (weighted mean of neighbors’ values), respectively [33]. In addition, all the values are standardized values rather than the original data.

In the scatterplots, the units in quadrant I and quadrant III exhibit positive spatial autocorrelation, whereas those in quadrant II and quadrant IV show negative spatial autocorrelation. In detail, quadrant I represents the high–high type spatial autocorrelation, which means the zones with high workforce density are surrounded by zones with high densities, and quadrants II, III, and IV represent the low–high type (zones with low health workforce density surrounded by zones with high densities), low–low type (zones with low health workforce density surrounded by zones with low densities) and high–low type (zones with high health workforce density surrounded by zones with low densities), respectively. Based on the four quadrants, all the administrative units can be divided into four categories, and the classification results show the status of each administrative unit in the space.

#### 3.3.3. Local Moran’s I

Local Moran’s I, which is also called a local indicator of spatial association (LISA), is a local indicator of variations within the study area [35]. Local Moran’s I is defined as

(4) $${\mathrm{Local}\;\mathrm{Moran}}^{\prime }s\;I=\frac{\left(x_{i}-\bar{x}\right)}{m_{0}}\underset{j}{\sum }W_{ij}\left(x_{j}-\bar{x}\right)\;m_{0}=\underset{i}{\sum }{\left(x_{i}-\bar{x}\right)}^{2}/n.$$

“The operation of summing *j* is limited to the surrounding areas of I” [32]. Commonly, it is used to identify outliers and leverage points, i.e., units with statistical significance, in each quadrant in the scatterplots [36]. In this study, the outliers and leverage points that display fairly unique characteristics have either a relatively abundant or an inadequate health workforce and should receive focused attention to promote equitable distribution. To visually display these units, we highlighted the locations for which Local Moran’s I passed the 95% significance level on China’s geographical map. In addition, all the locations were classified by the type of association, which corresponds to the four quadrants in Moran’s I scatterplots. As the highlighted areas show relatively significant correlations with adjacent areas, which are also known as spatial clusters, these maps are commonly referred to as univariate LISA cluster maps.

### 3.4. Software Tool

First, the Theil index coefficient is calculated using Microsoft Excel 2013 (Microsoft Corporation, Redmond, WA, USA). Then, the Global and Local Moran’s I and Moran’s I scatterplots are computed by utilizing GeoDa 1.8.14 (Center for Spatial Data Science in the University of Chicago, Chicago, IL, USA). Finally, thematic maps are developed with ArcGIS 10.0 (ESRI, Redlands, CA, USA).

## 4. Results

### 4.1. Dynamics of Health Workforce Density

Table 1 summarizes China’s health workforce density at the provincial level for doctors and nurses in 2004, 2009, and 2014. Two equal time sections (2004–2009 and 2009–2014) divided by the New Medical Reform were created. Through the efforts of the government, society, and the health system, China experienced a rapid increase in the density of doctors and nurses, which was evidenced by the steady growth rate.

The average annual growth rate of the density of doctors remained almost unchanged before and after the New Medical Reform, with 3.6% and 3.7% in the two subperiods, respectively. In comparison with other areas, the density of doctors in eastern administrative units increased more after than before the New Medical Reform, with the average annual growth rate rising from 2.7% to 4.1% after 2009. By contrast, Central and Western China experienced slower growth after the New Medical Reform (4.3% and 3.4% for central regions, and 4.0% and 3.7% for western regions).

In spite of the rapid growth nationwide, the distribution of the health workforce has been relatively unbalanced. Some administrative units in Eastern China, such as Beijing and Shanghai, presented densities almost twice than those in some southwest provinces in 2014.

The density of nurses also maintained rapid growth over the entire period, with an annual growth rate more than twice that of doctors. Moreover, the annual growth rate of the density of nurses dramatically accelerated after 2009, increasing from 6.8% to 8.7%. In comparison with doctors, all subregions experienced an increase in the density of nurses, as overwhelming progress was observed not only in Eastern China but also in Central and Western China. Furthermore, the densities of nurses in Central and Western China experienced more rapid growth than that of nurses in Eastern China in the second subperiod.

### 4.2. Theil Index of Health Workforce Distribution

The Theil index was adopted in this study to calculate the global equity in health workforce distribution for China and the contribution rate of China’s three subregions (the eastern, central, and western regions). The Theil index results shown in Table 2 indicate a continuous improvement in the equity of health resource distribution in China at the provincial level.

In particular, the Theil index for doctors decreased from 0.032 in 2004 to 0.021 in 2009 and 0.011 in 2014. The contribution rate between the three parts of China first decreased and then increased after the New Medical Reform. In terms of the regional divisions, the Theil index for Eastern China showed no significant difference from those in Western and Central China.

The Theil index for nurses gradually decreased from 0.048 in 2004 to 0.013 in 2014. The contribution rate within the three parts of China kept stable at first but increased after the New Medical Reform. Among the three regions, the Theil index for Eastern China was relatively higher than those in Western and Central China at both time points.

### 4.3. Global Spatial Autocorrelation

**Figure 2:**
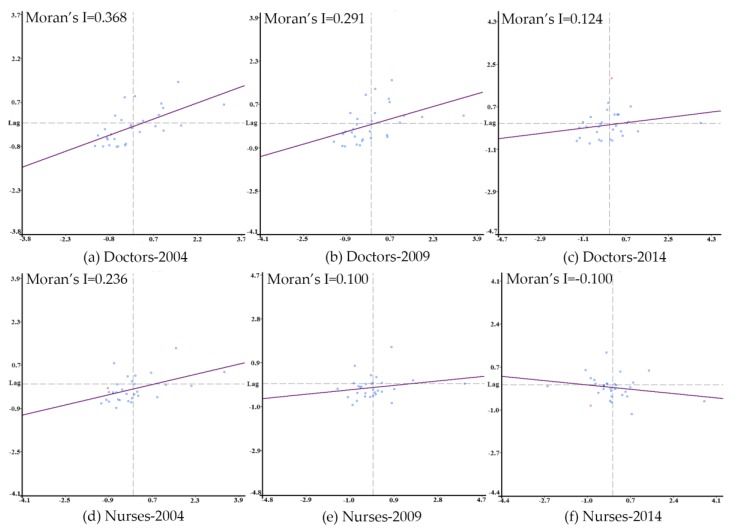
Moran’s I scatterplots for doctors and nurses in 2004, 2009, and 2014.

Moran’s I scatterplots and the values of Global Moran’s I are shown in Figure 2. The slope of the fitting line in each graph is the value of Global Moran’s I. Three time points, namely, 2004, 2009, and 2014, were selected to display the spatial autocorrelation of doctors and nurses. As shown, most administrative units were found in Quadrants I and III in 2004 for both doctors and nurses, suggesting a relatively strong positive spatial autocorrelation. From 2004 to 2014, the Global Moran’s I decreased by certain degrees for both doctors and nurses (especially for the latter), indicating a downward tendency of the positive spatial autocorrelation. Additionally, the number of points situated in Quadrants II and IV gradually increased during the entire period.

Specifically, by taking 2009 as the dividing point, the decrease of the Global Moran’s I for doctors accelerated after the New Medical Reform—the Global Moran’s I for doctors decreased by 0.077 in the first subperiod and by 0.167 in the second subperiod. Meanwhile, the Global Moran’s I for nurses decreased from 0.236 to 0.100 from 2004–2009 and then to −0.100 in the second subperiod. Collectively, the decline of Global Moran’s I indicates that the relatively serious concentration of similar values, which shows the spatial concentration of the density of doctors and nurses, gradually reduced.

### 4.4. Spatial Classification

Table 3 and Table 4 present the detailed spatial classifications of 30 administrative units for doctors and nurses, respectively. For doctors at all the time points, the administrative units of the high–high type (Quadrant I in scatterplots) were more situated in Eastern China, whereas the administrative units that show low–low type spatial autocorrelation (Quadrant III in scatterplots) were mainly distributed in the inland areas, especially in Southwest China, indicating the regional disparity of the health workforce distribution. Since 2004, the number of administrative units belonging to the low–low type decreased continually, from 16 in 2004 to 15 in 2009 and 10 in 2014. In addition, the number of administrative units belonging to high–high, low–high, and high–low groups increased by 1, 2, and 3 during the entire period, respectively.

With regard to nurses, the results were similar to those for doctors in terms of quantity changes. During the entire time period, the number of administrative units belonging to the high–high type and the low–low type decreased, whereas the number of administrative units belonging to the low–high and high–low types increased by 4 and 2, respectively. Nevertheless, significant changes were not observed in the spatial transformation of the four types before and after the New Medical Reform.

Overall, the spatial distribution characteristics of Eastern China were remarkably more complex than those in other areas for doctors and nurses among the three regions. The administrative units in Eastern China were relatively evenly distributed in the four quadrants, whereas the administrative units in Central and Western China were mainly distributed in one or two quadrants, especially Quadrant III (low–low type). For example, high–high type administrative units were not identified in Western China in terms of nurses at all the time points, whereas Eastern China covered all the types invariably.

In addition, as all the administrative units can be divided into the four types of spatial autocorrelation in Moran’s scatterplots, there are 12 (4 × 3) possible transitions between any two time points. Spatial stability, which indicates the relative stability of the health workforce distribution, can be calculated as the proportion of administrative units that remained unchanged [37]. Specifically, the values of spatial stability in 2004–2009 and 2009–2014 were 0.900 and 0.700 for doctors, and 0.700 and 0.633 for nurses, respectively.

### 4.5. Local Spatial Autocorrelation

**Figure 3:**
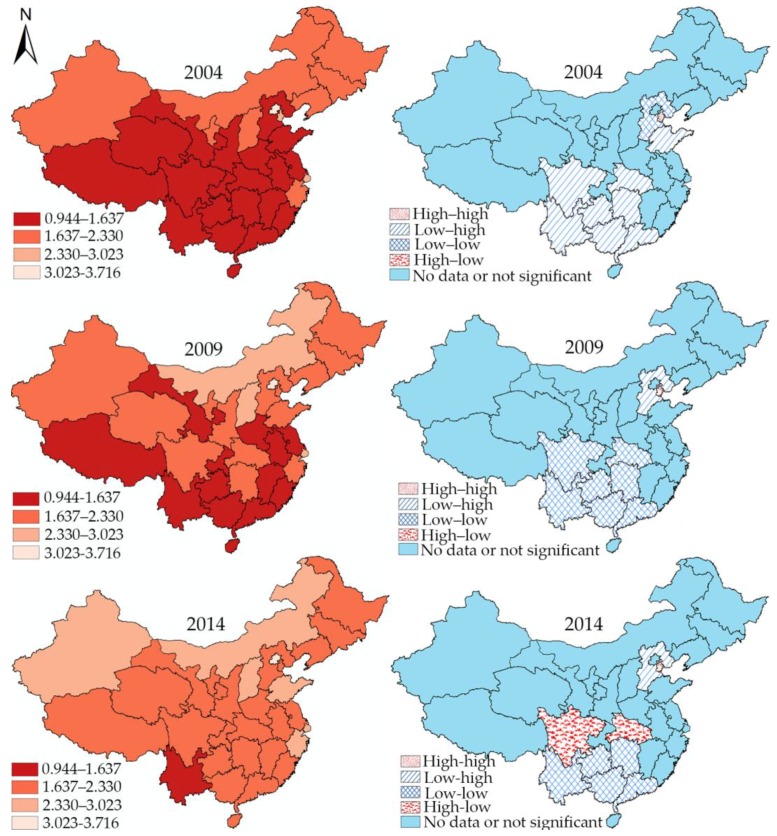
Hierarchical maps and univariate local indicator of spatial association (LISA) cluster maps of the density of doctors in mainland China in 2004, 2009, and 2014.

**Figure 4:**
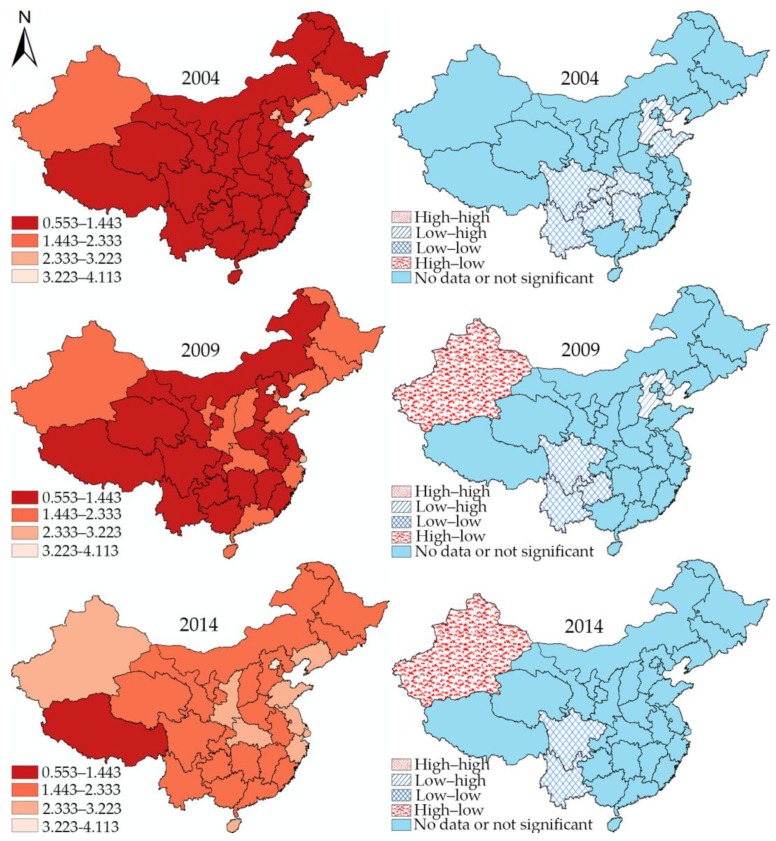
Hierarchical maps and univariate LISA cluster maps of the density of nurses in mainland China in 2004, 2009, and 2014.

Figure 3 and Figure 4 show the hierarchical maps and univariate LISA cluster maps for doctors and nurses, respectively, demonstrating the detailed spatial distribution and transformation. The four classes in the hierarchical maps are classified based on the maximum and minimum of health workforce density during 2004, 2009, and 2014. The darker the color is, the smaller is the health workforce density.

The univariate LISA cluster map view indicates that two subcategories of the health workforce have their own characteristics. For doctors in 2004, the low–low cluster units can be found all over the country. During the first subperiod, Shandong province moved out of the low–low cluster area, and Sichuan and Hubei turned from low–low cluster types into high–low cluster types in the second subperiod. In particular, the low–high and high–high cluster areas did not change over time. The low–high cluster type was located only in Hebei Province, whereas Tianjin was the only administrative unit showing a high–high cluster feature.

On the other hand, the complexity of cluster features for the density of nurses reduced over time. Shandong, Chongqing, Hubei, and Hunan moved out of the low–low cluster areas in the first subperiod, and Jiangxi followed in the second subperiod. In addition, Hebei Province exhibited low–high cluster feature only in 2004 and 2009, whereas the high–low cluster feature of Xinjiang was significant only at the last two time points.

## 5. Discussion

Geographical factors play an important role in contributing to inequity in health resource distribution. However, the spatial location information of each administrative unit is overlooked by the existing measuring methods. To fill the gap, this study makes initial efforts by combining traditional economic methods and spatial statistics to measure the equity in the health workforce distribution in China and provide policy recommendations to promote better health equity.

First, during the period from 2004 to 2014, the equity in the health workforce distribution of mainland China and its three parts continuously improved, accompanied by the rapid growth of the health workforce density. Divided into two periods by 2009, the New Medical Reform triggered a more rapid growth of the health workforce density and further promoted the improvement of the equity in the health workforce distribution, which is consistent with findings of other scholars (e.g., Liu et al., 2016). Evidently, the traditional economic method merely provides the overall situation of the equity in health workforce distribution, which limits its practical significance for supporting evidence-based policy making. In this regard, more evidence is needed.

Second, the spatial statistics reveal the spatial changing patterns of health workforce distribution before and after the New Medical Reform. In particular, the results of spatial stability indicate that the New Medical Reform helped disadvantaged province units veer away from disadvantages in health workforce distribution. Particularly at the local level, the changes of the spatial cluster areas also reflect the spatial changing patterns of health workforce distribution in China, and almost all the low-low cluster areas are concentrated in the central and southwestern parts of China. As previously mentioned, the provincial units displaying low–low cluster features gradually reduced and either became insignificant or turned into other types, while the elimination process of doctors and nurses show relatively different characteristics. For doctors, the low–low cluster areas gradually disappeared from north to south (Shandong in the first subperiod; Sichuan and Hubei in the second), while in contrast, the low–low cluster areas for nurses disappeared from east to west (Shandong, Hubei, Hunan and Chongqing in the first subperiod; Guizhou in the second). The results imply a knottier situation in southwest China. For instance, Yunnan province, exactly situated in the southwest border area, is the only low–low cluster overlap area for doctors and nurses in 2014 and is in urgent need of a larger health workforce. Altogether, the results imply that economic strength and geographic position play a vital role in influencing health workforce distribution. Compared to other regions, the eastern provincial units with high economic strength are unlikely to suffer health workforce shortage and are better positioned to cope with the shortage of the health workforce.

Finally, interactions between the traditional economic methods and spatial statistics are worth noting. First, because traditional economic methods and spatial statistics measure different dimensions of health workforce distribution, their results should be explained from an integrated perspective. More specifically, traditional economic methods measure the gaps among all the units, while spatial autocorrelation analysis is used to identify how these gaps are distributed on the map. A positive spatial autocorrelation indicates that the gaps usually exist between nonadjacent units, whereas a negative spatial autocorrelation suggests that the gaps tend to occur among adjacent units. Therefore, a positive spatial autocorrelation indicates a totally different situation compared with a negative one. In this study, the decreasing Theil index as well as Global Moran’s I for both doctors and nurses suggests gradual improvement in health equity in either the spatial cluster areas or others. However, the negative Global Moran’s I, meaning the concentration of dissimilar values, may imply a tendency of brain drain from adjacent areas for nurses and likely doctors in the future on the basis of current trends.

Second, the spatial statistics provide a geographic perspective and serve as the foundation to understand the results and trends of traditional economic methods. In a broad sense, the decrease of the Theil index for the entire region corresponds to the emigration of the administrative units in Quadrant III to other quadrants over time. From the sub-regional perspective, in most cases, the more administrative units are concentrated in certain quadrants, the lower the Theil index is. For instance, the higher Theil index of Eastern China for nurses can be explained by its complex spatial distribution characteristics. Specifically, the administrative units in Eastern China are relatively evenly distributed in the four quadrants, whereas the administrative units in Central and Western China are mainly distributed in one or two quadrants, especially Quadrant III (low–low type). Taking the numbers of doctors in 2009 as another example, the administrative units in Central China are situated in quadrants I and III only, whereas those in Western and Eastern China covered all four quadrants. Correspondingly, the Theil index for Central China is lower than that of the other two regions.

Overall, spatial statistics have unique advantages in studying the equity in health workforce distribution. While traditional economic methods measure the global equity level, spatial statistics can be used to explore the dynamics of health workforce distribution and identify the priority areas for health workforce allocation. The combination of the two analytical tools helps this study paint a better picture of human workforce distribution in China. We believe that spatial statistics can be used not only in studies on the equity in health workforce distribution but also in similar studies related to other health resources (financial, material, etc.). However, spatial statistics can only be used to explore the spatial disparities rather than identify possible causes.

As an ecological study, this study faces the risk of ecological fallacy. That is, in this study, a higher health workforce density in one region does not mean the higher health services availability for people in this region as medical service provision is affected by a lot of factors ((e.g., affordability, health workforce quality, medical facilities, etc.). Besides, due to the data accessibility, this study examined only the data at the provincial level, and it therefore cannot eliminate the scale problem, which is a classical issue in geography, namely, the modifiable areal unit problem (MAUP) [38]. To either minimize the problem of MAUP or provide more evidence for health workforce planning, research on health workforce distribution at the country or city level would be necessary. We also suggest future studies to incorporate more spatial indictors, e.g., measures of spatial segregation [39] or space-time scan statistics [40], into studying the spatial distribution of healthcare resources.

## 6. Conclusions

The introduction of emerging spatial statistics injects new energy into the enduring health equity issues, as the traditional economic methods and spatial statistics complement each other in understanding the equity in health workforce distribution. Simply put, the traditional economic methods reveal the overall equity trends, while the detailed spatial analysis presents the spatial changing patterns and lays a foundation for understanding the results of the traditional economic methods.

In the Chinese context, the New Medical Reform has increased the growth rate of the density of nurses. Meanwhile, the improvement of the global equity in health workforce distribution can also be observed after the New Medical Reform. However, health equity remains an unsolved challenge for China. This study sheds light on priority areas for health workforce allocation through the spatial-temporal transformation of health workforce distribution. However, balancing the distribution of the health workforce is a complex task that cannot be achieved from within the health sector or in the short term. Instead, it requires joint efforts from government agencies (e.g., health, economic and human resources sectors) and coherent consideration across all policies and regional cooperation in disadvantaged areas to prevent the brain drain of the health workforce. Additionally, owing to the long training cycle of the health workforce, long-term planning is necessary to ensure the quantity and quality of the health workforce in disadvantaged areas. The continual development of the health workplace is crucial because it equips health providers with the appropriate skill mix, which is important in health service delivery.

## Figures and Tables

**Table 1:** Growth of the density of doctors and nurses in China.

Region	Density of Doctors ^1^			Density of Nurses			Average Growth Rate of Doctors		Average Growth Rate of Nurses	
	2004	2009	2014	2004	2009	2014	2004–2009	2009–2014	2004–2009	2009–2014
Eastern China	1.64	1.88	2.30	1.19	1.58	2.37	2.7%	4.1%	5.8%	8.4%
Beijing (BJ)	3.29	3.58	3.72	2.78	3.52	4.11	1.7%	0.7%	4.8%	3.2%
Tianjin (TJ)	2.46	2.25	2.20	1.91	1.88	2.08	−1.8%	−0.4%	−0.3%	2.1%
Hebei (HeB)	1.48	1.76	2.14	0.79	1.06	1.65	3.5%	3.9%	6.2%	9.2%
Liaoning (LN)	2.19	2.20	2.31	1.73	1.95	2.41	0.1%	1.0%	2.4%	4.4%
Shanghai (SH)	2.51	2.40	2.52	2.19	2.37	2.96	−0.9%	1.0%	1.6%	4.6%
Jiangsu (JS)	1.43	1.60	2.24	1.04	1.42	2.37	2.4%	6.9%	6.5%	10.8%
Zhejiang (ZJ)	1.76	2.16	2.65	1.16	1.67	2.64	4.2%	4.2%	7.5%	9.5%
Fujian (FJ)	1.24	1.50	1.98	0.93	1.31	2.25	3.9%	5.7%	7.0%	11.5%
Shandong (SD)	1.51	1.86	2.36	1.05	1.47	2.51	4.3%	4.9%	7.1%	11.2%
Guangdong (GD)	1.36	1.59	2.02	1.11	1.51	2.18	3.2%	4.9%	6.4%	7.5%
Central China	1.38	1.70	2.01	0.94	1.33	2.06	4.3%	3.4%	7.2%	9.1%
Shanxi (SX)	2.05	2.47	2.46	1.23	1.67	2.17	3.8%	−0.1%	6.3%	5.3%
Jilin (JL)	2.15	2.20	2.30	1.46	1.55	2.09	0.4%	0.9%	1.2%	6.2%
Heilongjiang (HLJ)	1.68	1.94	2.12	1.17	1.46	2.03	3.0%	1.8%	4.5%	6.8%
Anhui (AH)	1.00	1.38	1.71	0.70	1.14	1.83	6.7%	4.3%	10.4%	9.9%
Jiangxi (JX)	1.17	1.34	1.64	0.83	1.21	1.85	2.7%	4.2%	7.9%	8.9%
Henan (HeN)	1.13	1.59	2.01	0.77	1.18	2.03	7.2%	4.7%	9.0%	11.4%
Hubei (HuB)	1.49	1.71	2.17	1.10	1.53	2.48	2.8%	4.9%	6.8%	10.2%
Hunan (HuN)	1.34	1.65	1.98	0.86	1.30	2.02	4.2%	3.7%	8.5%	9.3%
Western China	1.36	1.66	1.99	0.85	1.21	2.12	4.0%	3.7%	7.4%	11.8%
Neimenggu (NMG)	2.10	2.82	2.48	1.11	1.44	2.26	6.0%	−2.5%	5.3%	9.5%
Guangxi (GX)	1.09	1.38	1.82	0.89	1.29	2.19	4.7%	5.8%	7.9%	11.1%
Chongqing (CQ)	1.17	1.56	1.94	0.65	1.12	2.10	5.9%	4.5%	11.4%	13.5%
Sichuan (SC)	1.30	1.69	2.21	0.70	1.11	2.16	5.5%	5.4%	9.8%	14.1%
Guizhou (GZ)	0.94	1.17	1.65	0.55	0.90	1.92	4.4%	7.1%	10.3%	16.2%
Yunnan (YN)	1.21	1.32	1.60	0.83	1.01	1.76	1.9%	3.9%	4.1%	11.6%
Xizang (XZ)	1.59	1.53	1.76	0.67	0.68	0.85	−0.7%	2.8%	0.0%	4.8%
Shaanxi (SXX)	1.61	1.87	2.03	1.01	1.47	2.58	3.0%	1.6%	7.8%	11.8%
Gansu (GS)	1.32	1.47	1.84	0.85	1.04	1.75	2.3%	4.5%	4.1%	11.0%
Qinghai (QH)	1.60	1.84	2.22	1.15	1.41	2.19	2.8%	3.9%	4.1%	9.2%
Ningxia (NX)	1.82	1.93	2.27	1.21	1.58	2.28	1.2%	3.2%	5.5%	7.7%
Xinjiang (XJ)	2.19	2.17	2.38	1.57	1.89	2.60	−0.3%	2.0%	3.7%	6.6%
SUM	1.47	1.76	2.11	1.01	1.40	2.12	3.6%	3.7%	6.8%	8.7%

^1^ Unit: Number per 1000 persons.

**Table 2:** Theil index of health workforce allocation in 2004, 2009, and 2014.

Categories	Year	Theil Index				Contribution Rate	
		Total	Western China	Central China	Eastern China	Within Groups	Among Groups
Doctors	2004	0.032	0.028	0.029	0.028	87.5%	12.5%
	2009	0.021	0.026	0.016	0.019	90.5%	9.5%
	2014	0.011	0.010	0.007	0.009	81.8%	18.2%
Nurses	2004	0.048	0.034	0.026	0.049	77.1%	22.9%
	2009	0.027	0.020	0.008	0.032	77.8%	22.2%
	2014	0.013	0.010	0.004	0.018	84.6%	15.4%

**Table 3:** Classification of four types of spatial autocorrelation for doctors.

Year	Type	Number	Western China	Central China	Eastern China
2004	High–high	7	NX, NMG	HLJ, JL	BJ, LN, TJ
	Low–high	3	GS	none	HeB, JS
	Low–low	16	GX, CQ, SC, GZ, YN, XZ, SXX, QH	HeN, HuB, AH, HuN, JX	SD, FJ, GD
	High–low	4	XJ	SX	SH, ZJ
2009	High–high	10	SXX, NX, NMG	JL, SX, HLJ	TJ, SH, BJ, LN
	Low–high	3	GS	none	JS, HeB
	Low–low	15	QH, GZ, GX, SC, YN, CQ, XZ	HeN, AH, JX, HuN, HuB	FJ, GD, SD
	High–low	2	XJ	none	ZJ
2014	High–high	8	NMG	JL, SX	JS, SH, TJ, BJ, LN
	Low–high	5	GS, SXX	AH, HLJ	HeB
	Low–low	10	XZ, GZ, YN, GX, CQ,	HeN, JX, HuN	GD, FJ
	High–low	7	NX, XJ, QH, SC	HuB	SD, ZJ

**Table 4:** Classification of four types of spatial autocorrelation for nurses.

Year	Type	Number	Western China	Central China	Eastern China
2004	High–high	5	none	HLJ, JL	BJ, TJ, ZJ
	Low–high	3	NMG	none	HeB, JS
	Low–low	16	GX, CQ, SC, GZ, YN, XZ, SXX, GS	HeN, HuB, AH, HuN, JX	SD, FJ, GD
	High–low	6	XJ, NX, QH	SX	SH, LN
2009	High–high	4	none	JL	TJ, SH, ZJ
	Low–high	5	GS, NMG	HLJ	JS, HeB
	Low–low	12	QH, GZ, GX, SC, YN, CQ, XZ	HeN, AH, JX, HuN	FJ
	High–low	9	XJ, SXX, NX	HuB, SX	BJ, SD, GD, LN
2014	High–high	4	none	none	JS, SH, FJ, ZJ
	Low–high	7	GS, CQ	AH, JL, JX	HeB, TJ
	Low–low	11	XZ, GZ, YN, GX, SC, QH	HeN, HLJ, HuN, SX	GD
	High–low	8	NX, XJ, NMG, SXX	HuB	SD, BJ, LN

## Supplementary Materials

The following are available online at http://www.mdpi.com/1660-4601/15/7/1309/s1, Table S1. The health workforce and population data in 2004, 2009, and 2014.
